# Soluble Interleukin-2 Receptor Is a Promising Serum Biomarker for Granulomatous Disease in Common Variable Immune Deficiency

**DOI:** 10.1007/s10875-020-00947-8

**Published:** 2021-01-06

**Authors:** Astrid C. van Stigt, Virgil A. S. H. Dalm, Nicole M. A. Nagtzaam, Damian A. van Rijswijk, Barbara H. Barendregt, P. Martin van Hagen, Hanna IJspeert, Willem A. Dik

**Affiliations:** 1 Department of Immunology, Laboratory Medical Immunology, Erasmus University Medical Center, Rotterdam, the Netherlands; 2 Department of Internal Medicine, Division of Clinical Immunology, Erasmus University Medical Center, Rotterdam, the Netherlands; 3 Academic Center for Rare Immunological Diseases (RIDC), Erasmus University Medical Center, Rotterdam, the Netherlands

To the editor:

Common variable immune deficiency (CVID) is a primary antibody deficiency characterized by a marked decrease of immunoglobulin (Ig) G in combination with low IgA and/or IgM, an impaired response to immunization and recurrent infections [1]. Up to 68% of CVID patients have additional noninfectious complications (NIC), including autoimmune complications, malignancies, and granulomatous disease. These NIC are associated with increased morbidity and mortality [2–4]. Granulomatous disease, especially granulomatous and lymphocytic interstitial lung disease (GLILD), leads to a significant reduction in median survival in CVID patients [5]. Granuloma formation is thought to be initiated by CD4+ T lymphocytes that become activated after interaction with antigen presenting cells [6]. Activated CD4+ T lymphocytes secrete cytokines that subsequently stimulate macrophage activation and tumor necrosis factor (TNF)-α production, ultimately leading to the characteristic immune cell agglomerates (i.e., granulomas) in various organs.

Several studies have tried to identify potential biomarkers for NIC in CVID. It has been shown that increased numbers of PD-1 high CCR7 low CD4+ T follicular-helper-lymphocytes are associated with autoimmune complications or granulomatous disease [7]. Furthermore, an association between elevated serum IgM and interstitial lung disease in CVID, which was related to B cell follicles within the lung parenchyma, is reported [8]. In pediatric CVID patients, it was observed that reduction in total B and NK lymphocytes with an increase in CD8+ T lymphocytes was associated with presence of bronchiectasis [9]. Hartono et al. [10] described a prediction model for the presence of GLILD in CVID. They showed that a medical history of splenomegaly, immune thrombocytopenia (ITP) and/or autoimmune hemolytic anemia (AIHA), IgA levels < 13 mg/dl, and CD21-low B lymphocytes > 5% of total CD21 B cells are to be predictive measures for GLILD in CVID [10]. We were interested to see whether these clinical and immunological features were also predictive for GLILD in our CVID cohort. Therefore, we collected data on the presence of splenomegaly, ITP, AIHA, the IgA levels, and the frequency of CD21-low B lymphocytes from our cohort of CVID patients, which resulted in a subgroup of 38 CVID patients of which all these characteristics were available. In our cohort, only the presence of splenomegaly was found to be significantly increased in patients with GLILD when compared to CVID patients without GLILD. Moreover, also in patients with granulomatous complications affecting other organ systems, including spleen and lymph nodes, only splenomegaly positively correlated with the presence of granulomatous disease (Supplementary Table 1). However, it has to be taken into account that sample sizes used for analysis were small.

These findings prompted us to search for a low-invasive biomarker to diagnose and monitor the progression of granulomatous disease in CVID. The soluble form of the interleukin-2 receptor (sIL-2R or sCD25), which is secreted by activated T lymphocytes, is frequently used to monitor immune cell activation. Elevated sIL-2R levels have been reported in various pathological conditions, including autoimmune diseases, infection, and malignancies [11–14]. Previous studies showed that sIL-2R levels in CVID patients in general are higher than in healthy controls (HCs) [15–17]. Moreover, it has been demonstrated that sIL-2R levels are higher in CVID patients with NIC when compared to patients with infections-only (IO), or CVID patients with gastrointestinal symptoms [18, 19]. Furthermore, a decline of sIL-2R was found to coincide with clinical improvement after abatacept treatment in patients with CTLA-4 haploinsufficiency and lipopolysaccharide responsive beige-like anchor protein (LRBA)-deficiency [7, 20, 21].

The relation between sIL-2R level and granulomatous disease in CVID patients has not been extensively studied. Only one case study described a decline of serum sIL-2R in a CVID patient with granulomatous lung diseases after effective immunosuppressive therapy with azathioprine and rituximab [22]. In sarcoidosis, an inflammatory multisystem granulomatous disease of unknown etiology, serum sIL-2R, was reported as a sensitive biomarker [23, 24]. Therefore, we aimed to determine whether serum sIL-2R level can be used as a low-invasive biomarker for detection of granulomatous disease and for monitoring granuloma progression or remission in CVID patients.

To this aim we performed a retrospective single-center analysis, evaluating serum sIL-2R levels in 48 CVID patients, including 12 patients with granulomatous disease, 13 healthy controls (HC), and 79 sarcoidosis patients previously reported by our group (Supplemental Table 2 and the method section in the supplemental data) [23]. Similar to the previous studies, the CVID group displayed significantly higher sIL-2R levels (median sIL-2R 4,539 pg/ml; range: 1,037–48,875 pg/ml) compared to the HC group (median sIL-2R 1,419 pg/ml; range: 1,096–3,328 pg/ml) (Supplemental Fig. 1a, Supplemental Table 2) [15, 17, 18]. The variability of sIL-2R levels observed within the CVID group was not related to the type of immunoglobulin replacement therapy, since no significant difference was observed in sIL-2R levels across the various treatment modalities (Supplemental Fig. 1b). Interestingly, the group of CVID patients with NIC (CVID+NIC, *n* = 22) had significantly higher sIL-2R levels (median: 6,612 pg/ml; range: 1,620–48,875 pg/ml) than the CVID group with IO (CVID IO; *n* = 26; median sIL-2R: 2,918 pg/ml; range: 1,037–17,300 pg/ml) or HC (Supplemental Fig. 1c, Supplemental Tables 2 and 3). This is in line with a previous study that showed significantly higher sIL-2R levels in CVID patients with NIC, compared to CVID patients with infections only or HC [18].

To determine whether CVID patients with granulomatous disease have increased levels of sIL-2R, we compared sIL-2R levels prior to and at the time when there was reported progression of granulomatous disease (CVID+p.granuloma) with CVID patients with IO. Data on progression of granulomatous disease were derived from clinical reports, computed tomography (CT) scan, magnetic resonance imaging (MRI) or pathology reports from patient files. Interestingly, the CVID+granuloma group (median sIL-2R: 10,853 pg/ml; range: 4,458–13,049 pg/ml) displayed significantly higher serum sIL-2R levels than CVID IO (Fig. 1a). Moreover, sIL-2R levels increased upon progression of granulomatous disease (CVID+p.granuloma, median sIL-2R: 22,274 pg/ml; range: 5,476–48,875 pg/ml) (Fig. 1a). Also, median levels of sIL-2R in both CVID+granuloma and CVID+p.granuloma were higher than the median levels we previously measured in sarcoidosis patients (*n* = 79; median sIL-2R: 6,000 pg/ml; range: 1,600–90,300 pg/ml), only reaching statistical significance for CVID+p.granuloma (Fig. 1a, Supplemental Table 2) [23].

**Fig. 1:**
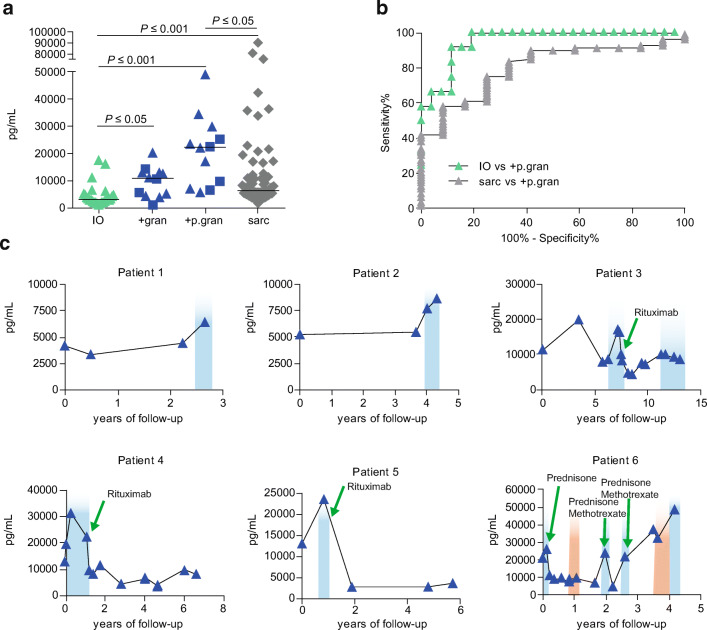
Discriminative capacity of serum sIL-2R. **a** sIL-2R levels per subgroups of CVID (CVID IO (IO, *n =* 26); CVID+granuloma (+gran, *n* = 12); CVID+progression of granulomatous disease (+p.gran, *n =* 12) and sarcoidosis (sarc, *n* = 79). **b** ROC-curves showing discriminating capacity of sIL-2R. Black lines indicate median, square symbols are CVID patients with multiple NIC. **c** Longitudinal sIL-2R analysis of 6 CVID+granuloma patients. sIL-2R levels are showed per years after start sampling (first time point) of sIL-2R. Blue gradient indicates progression of granulomatous disease derived from patient files. Green arrows shown in patient 3 until 6 indicate start of antigranuloma treatment. The red gradient indicate episodes of collagen colitis in patient 6

To further determine whether serum sIL-2R could be used to differentiate between CVID patients with progression of granulomatous complications and other CVID patients, a Receiver Operator Curve (ROC) analysis was performed (Fig. 1b, Supplemental Fig. 1d). This revealed that serum sIL-2R can be used to differentiate between CVID+p.granuloma and CVID IO (area under the curve (AUC) = 0.95) (Fig. 1b, Supplemental Table 4). Using a cutoff value for sIL-2R of > 6,376 pg/ml, yielded a high sensitivity of 91.7% and a high specificity of 88.5% with a Youden’s Index (YI) of 0.80 to differentiate between CVID+p.granuloma and CVID IO. These findings suggest that high sIL-2R level (> 6,376 pg/ml) might represent a clinically valuable biomarker to differentiate CVID patients with granulomatous complications from other CVID patients.

The clinical context of the patient of which serum sIL-2R level is measured and interpreted is important, since sIL-2R is increased in many inflammatory diseases or complications [14]. Therefore, the sensitivity is predicted to be high, while specificity is low. To evaluate whether an increase of sIL-2R levels was associated with other inflammatory complications, clinical profiles of the included patients were studied, focusing on additional inflammatory complications, including presence of bronchiectasis, ground glass lesions, lymphoproliferation, splenomegaly, and intestinal complications, at time of serum sampling. No association was observed between other inflammatory complications and sIL-2R levels (Supplemental Table 3).

Although overlap existed in sIL-2R levels between sarcoidosis and CVID+p.gran, in general, sIL-2R levels in sarcoidosis were lower (median 6,000 pg/ml) than in CVID+p.gran (median 22,274 pg/ml) (Supplementary Table 2). We observed that serum sIL-2R could differentiate between CVID+p.granuloma and sarcoidosis with a sensitivity of 91.1%, specificity of 41.7% and YI from 0.33 using a cutoff value of < 23,223 pg/ml. This indicated that sIL-2R levels lower than 23,223 pg/ml are associated with lower probability of having granulomatous disease progression in CVID, but rather may be indicative for sarcoidosis, a granulomatous disease CVID patients can be misdiagnosed with. Therefore, additional diagnostics such as serum immunoglobulin levels should be performed to rule out the diagnosis of sarcoidosis [25].

Since we observed increasing sIL-2R levels upon progression of granulomatous disease (Fig. 1a), we further analyzed longitudinal serum sIL-2R measurements of six CVID patients with granulomatous disease of which longitudinal data were available. Interestingly, sIL-2R levels increased in all patients when progression of granulomatous disease was clinically observed (Fig. 1c). Moreover, a decrease in sIL-2R levels was observed after effective treatment with rituximab, prednisone, or prednisone in combination with methotrexate (Fig. 1c). This further strengthens the previous notion that serum sIL-2R can be used to monitor the effect of treatment for granulomatous disease in CVID [22]. In patient 6, the sIL-2R levels also increased during a second episode of collagen colitis, which supports the previous findings of increased sIL-2R levels in patient with gastrointestinal symptoms [19].

Of note, the baseline levels of sIL-2R levels were different for each CVID patient. This suggests that it is important to regularly monitor sIL-2R levels in order to timely detect an increase in serum sIL-2R relative to the patient baseline sIL-2R level. Although sIL-2R is related to many immune-regulated processes, an increase could reflect development or progression of granulomatous disease.

In summary, our data indicate that in patients with CVID sIL-2R levels rise with progression of granulomatous disease. On the other hand, sIL2R levels decline upon remission of granulomatous disease after treatment. These observations suggest that sIL-2R levels can be used as monitoring tool for evaluation of progression and treatment efficacy of granulomatous disease in CVID.

## Supplementary Information

ESM 1 (DOCX 291 kb)
