# Supplementary material for: Soluble Interleukin-2 Receptor Is a Promising Serum Biomarker for Granulomatous Disease in Common Variable Immune Deficiency
Source: J Clin Immunol. 2021 Jan 6;41(3):694–7. doi: 10.1007/s10875-020-00947-8 (PMC7921039; doi:10.1007/s10875-020-00947-8)
Supplement: Supplementary file 1 — (DOCX 291 kb) [file 10875_2020_947_MOESM1_ESM.docx]

**Soluble interleukin-2 receptor is a promising serum biomarker for granulomatous disease in common variable immune deficiency**

**Journal of Clinical Immunology, supplemental data**

Astrid C. van Stigt,^1,2,3^ Virgil A.S.H. Dalm,^1,2,3^ Nicole M.A. Nagtzaam,^1,3^ Damian A. van Rijswijk,^1,3^ Barbara H. Barendregt,^1,3^ P. Martin van Hagen,^1,2,3^ Hanna IJspeert,^1,3#^ Willem A. Dik^1,2,3#*^

# authors contributed equally

^1^ Department of Immunology, Laboratory Medical Immunology, Erasmus University Medical Center, Rotterdam, the Netherlands.

^2^ Department of Internal Medicine, Division of Clinical Immunology, Erasmus University Medical Center, Rotterdam, the Netherlands.

^3^Academic Center for Rare Immunological Diseases (RIDC), Erasmus University Medical Center, Rotterdam, the Netherlands.

Corresponding author:

Willem A. Dik, PhD

Erasmus MC

Dept. of Immunology

Dr. Molewaterplein 40

3015 GD Rotterdam

The Netherlands

Tel:+31-10-7043528

e-mail: w.dik@erasmusmc.nl

**Methods**

## *CVID patients and healthy controls*

Sera were obtained from 48 CVID patients (median age 45, range 20-80 years, male *n* = 19, female *n* = 29, collected between 08-11-1993 and 05-02-2020; details are summarized in Table 1) and 13 HCs (median age 54, range 28-64 years, male *n* = 4, female *n* = 9). CVID was diagnosed using the ESID criteria [1]. The study was approved by the medical ethical committee of Erasmus MC (MEC-2013-026, MEC-2016-606, MEC-2016-202). CVID characteristics (age at onset of symptoms, age at official diagnosis CVID, age at measurement sIL-2R, diagnostic delay, follow-up status, monogenetic screening) were obtained from patient files. In a small subset of CVID patients (n = 7), gene panel testing using whole exome sequencing (WES) was performed. Over 250 PID-associated genes were analyzed, based on the IUIS classifications 2015 [26], 2017 [27] and 2019 [28,29]. Corresponding versions of PID panel screening reported in supplemental table 3 can be found on https://www.erasmusmc.nl/nl-nl/patientenzorg/laboratoriumspecialismen/klinische-genetica. Agilent Sureselect Clinical Research Exome V2 Capture Enrichment kit (Agilent Technologies) and paired-end sequenced on the Illumina Hiseq platform (GenomeScan, Leiden, the Netherlands) were used for DNA exome enrichment, obtaining an average coverage of the exome ~50X. Reads were mapped to the genome with the BWA-MEM algorithm (http://bio-bwa.sourceforge.net/) and the Genome Analysis Toolkit HaplotypeCaller (http://www.broadinstitute.org/gatk/) was used for variant calling. Detected variants in the PID-associated genes were filtered and annotated with the Cartagenia software package and classified with Alamut Visual.

## *Categorization of CVID patients*

Patient files were analyzed to categorize patients according to comorbidities (see Table 1 for overview). CVID patients (*n* = 48) were categorized in CVID patients with infectious complications only (CVID IO, *n* = 26) and CVID patients with additional non-infectious complications (CVID+NIC, *n* = 22). The CVID+NIC included; 1) CVID patients with additional autoimmune complications but without granuloma (CVID+AI, *n* = 7), and 2) CVID patients with probable AI and definitely granulomatous disease (CVID+granuloma and CVID+p.granuloma, *n* = 12), or 3) malignancies without other non-infectious complications (CVID+malignancy, *n* = 3). Regarding malignancies, overall 6 patients had a reported malignancy in their medical history, of which three had only infections (CVID-malignancies group). One CVID+AI patient and two CVID+granuloma patients had also a malignancy. All CVID patients suffered from infectious complications in varying degrees.

## *Complications in CVID patients*

Complications (autoimmune complications, granuloma formation, malignancies) were derived from patients’ files. The time point of granulomatous complications (either initial development or granuloma progression, considered as increased granuloma disease activity) was obtained from patient files (reports, radiological examination, pathology reports). In 9 of 12 CVID+granuloma patients, granulomatous disease was proven by histopathological examination.

## *Serum sIL-2R measurements*

Serum sIL-2R measurements were performed (ELISA; Diaclone, Besancon Cedex, France) at the diagnostic facility of the Laboratory Medical Immunology (LMI) at Erasmus MC under strict quality procedures (ISO15189)). In addition, if available we retrieved sIL-2R from patients files for longitudinal analysis. All sIL-2R measurements were from serum samples obtained after official CVID diagnosis was made, thus during follow-up of the patient. Within the Erasmus MC, sIL-2R levels above 2500 pg/mL are considered elevated, based on serum sIL-2R levels measured in a cohort consisting of 101 healthy donors. These serum sIL-2R measurements were part of regular clinical care and also measured by the same diagnostic facility (LMI) at Erasmus MC using the same validated ELISA system. For all CVID patients and HCs, sIL-2R level from the first collected serum sample was used for analysis. Additionally, for CVID+p.granuloma patients dataset, sIL-2R levels were selected that were obtained within 6 months of increased granuloma disease activity. These time points were considered as relevant within this subcategory of CVID patients. In addition, the serum sIL-2R levels obtained from the CVID cohort where compared to our previously reported cohort of 79 histology proven sarcoidosis patients (median age 52, range. 25-83 years, male *n* = 40, female *n* = 39)[22]. By use of the date on which sIL-2R samples were collected, patient files were screened to assess if and which immunoglobulin replacement therapy was administered to the patient at time of sIL-2R sampling.

## *Baseline characteristics collection*

The patient baseline characteristics based on the study by Hartono *et al.*, being reported ITP/AIHA in medical history, splenomegaly in medical history, IgA levels < 13 mg/dl, and CD21lowBcells > 5%, were collected from the 48 CVID patient [9]. For IgA and CD21low B cell values, first reported time point was used. Splenomegaly was objectified based on radiological examination reports, considering splenomegaly as present when the spleen was described as > 11 cm or the terms ‘splenomegaly’ or ‘enlarged spleen’ were used. Eventually, of 38 out of 48 CVID patients, these characteristics were available.

## *Statistical analysis*

The statistical analyses were all performed in Graphpad Prism V7.0. Data was considered as nonparametric. For single comparison, *Mann-Whitney test* was performed. For multiple comparison analysis, *Kruskal-Wallis* with *Dunn* post-hoc correction was performed. Logistic regression analysis was performed using R Core Team (2013) (R: A language and environment for statistical computing. R Foundation for Statistical Computing, Vienna, Austria, URL <http://www.R-project.org/>)

References

26. Coopmans EC, Chunharojrith P, Neggers SJCMM, van der Ent MW, Swagemakers SMA, Hollink IH, et al. Endocrine disorders are prominent clinical features in patients with primary antibody deficiencies. Front Immunol. 2019;10:2079.

27. Bousfiha A, Jeddane L, al-Herz W, Ailal F, Casanova JL, Chatila T, et al. The 2015 IUIS phenotypic classification for primary immunodeficiencies. J Clin Immunol. 2015;35(8):727–38.

28. Bousfiha A, Jeddane L, Picard C, Ailal F, Bobby Gaspar H, al-Herz W, et al. The 2017 IUIS phenotypic classification for primary immunodeficiencies. J Clin Immunol. 2018;38(1):129–43.

29. Tangye SG, al-Herz W, Bousfiha A, Chatila T, Cunningham-Rundles C, Etzioni A, et al. Human inborn errors of immunity: 2019 update on the classification from the International Union of Immunological Societies Expert Committee. J Clin Immunol. 2020;40(1):24–64.

30. Picard C, al-Herz W, Bousfiha A, Casanova JL, Chatila T, Conley ME, et al. Primary immunodeficiency diseases: an update on the classification from the International Union of Immunological Societies Expert Committee for primary immunodeficiency 2015. J Clin Immunol. 2015;35(8):696–726.

**Supplemental Figure 1**

**
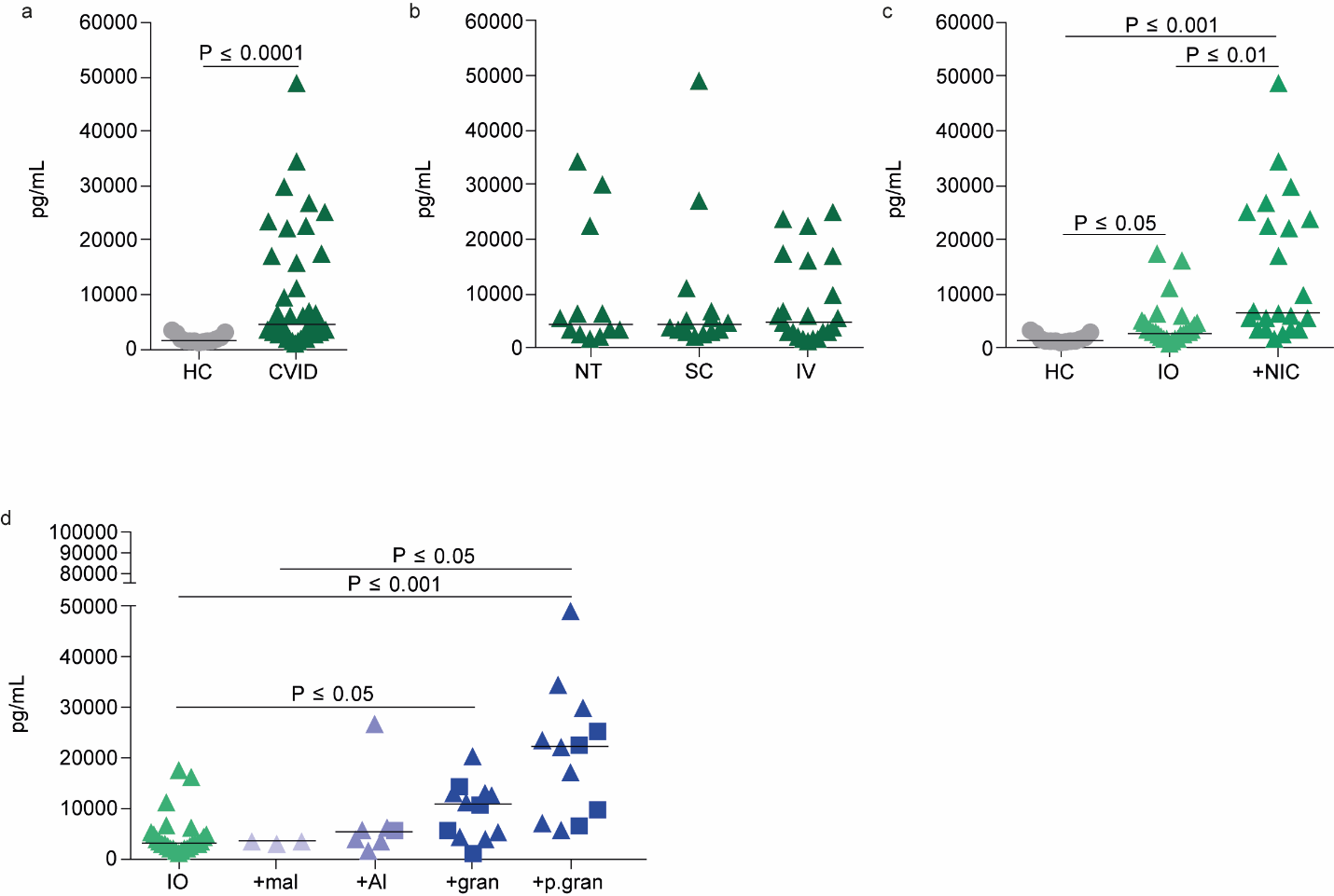
**

**a.** Increased sIL-2R CVID patients (*n* = 48) versus HC (*n* = 13). **b.** No effect at time of sIL-2R sampling was observed regarding no treatment (NT, *n* = 12 ) or receiving immunoglobulin replacement therapy subcutaneously (SC, *n* = 14) or intravenously (IV, *n* = 22).**c.** Increased sIL-2R levels in CVID+non-infectious complications (NIC, *n* = 22) versus CVID infections only (IO, *n* = 26) and HC (*n* = 13). **d**. sIL-2R levels per subgroups of CVID (CVID IO (IO, *n=* 26); CVID+malignancy (+mal, *n* = 3); CVID+AI (+AI, *n* = 7); CVID+granuloma (+gran, *n* = 12); CVID+progression of granulomatous disease (+p.gran, *n =* 12). Squared symbols indicate that the patients have more than one NIC.

# TABLES

**Supplementary Table 1. sIL-2R and baseline characteristics of 38 CVID patients**

|  | sIL2R (cutoff  > 6213 pg mL^-1^ | Splenomegaly | ITP/AIHA | IgA < 13 mg/dl | CD21low > 5% |
| --- | --- | --- | --- | --- | --- |
| p-value | 0.0004 | 0.0002 | n.s. | n.s. | n.s. |
| Sensitivity | 100 % | 100 % | 14.3 % | 85.7 % | 100 % |
| Sensitivity 95%CI | 59 to 100 % | 59 to 100 % | 0.4 to 57.9 % | 42.1 to 99.6 % | 59 to 100 % |
| Specificity | 83.3% | 76.7% | 86.7% | 50% | 36.7% |
| Specificity 95%CI | 65.3 to 94.4 % | 57.7 to 90.1 % | 69.3 to 96.2 % | 31.3 to 68.7 % | 19.9 to 56.1 % |
| Likelihood ratio | 6 | 4.29 | 1.07 | 1.71 | 1.58 |
| Youden's index | 0.83 | 0.77 | 0.01 | 0.36 | 0.37 |

**Supplemental table 2. Overview of HCs, CVID categories and sarcoidosis**

|  | Healthy control | CVID+  Infectious complications only | CVID  +autoimmune disease | CVID  +p.granuloma | CVID  +malignancy | Sarcoidosis |
| --- | --- | --- | --- | --- | --- | --- |
| Number | 13 | 26 | 7 | 12 | 3 | 79 |
| % female | 69.2 % | 57.7 % | 57.1 % | 75 % | 33.3 % | 50.6 % |
| % male | 30.8 % | 42.3 % | 42.9 % | 25 % | 66.7 % | 49.4 % |
| Median age  (range) years | 54 (28-64) | 44 (20-80) | 32 (24-77) | 51 (22-79) | 64 (53-79) | 52 (25-83) |
| Biopsy proven |  |  |  | 9 (75 %) |  | 79 (100 %) |
| Median sIL2R (range) pg mL^-1^ | 1,419  (1,096-3,328) | 2,918  (1,037-17,300) | 5,400  (1,620-26,800) | 22,274  (5,476-48,875) | 3,479  (2,765-3,488) | 6,000  (1,600-90,300) |
| % > 2500 pg mL^-1^ (within category) | 23.1 % | 73.1 % | 85.7 % | 100 % | 100 % | 97.5 % |

**Supplemental table 3. Characteristics and sIL-2R level of NIC CVID patients**

| Patient number | Complication group | Granuloma location | Granuloma biopsy proven | Type AI | Type malignancy | sIL-2R (pg/ml) | Age  onset symptoms | Age official CVID diagnosis | Age sIL-2R measurement | Diagnostic delay | Monogenetic screening performed | Monogenetic screening result | Additional complications |
| --- | --- | --- | --- | --- | --- | --- | --- | --- | --- | --- | --- | --- | --- |
| 1 | Granuloma | GLILD | no |  |  | 6,464 | 59 | 61 | 61 | 2 | no |  | no |
| 2 | Granuloma | Lung, diffusely spread lymphadenopathy | yes |  |  | 5,476 | 28 | 38 | 40 | 10 | no |  | Ground glass lesions |
| 3 | Granuloma | Lung, granulomatous thyroiditis | yes | IBD |  | 16,900 | 25 | 28 | 32 | 3 | no |  | lymphadenopathy |
| 4 | Granuloma | Cerebral, GLILD | yes | ITP |  | 22,148 | 16 | 16 | 16 | 0 | no |  | no |
| 5 | Granuloma | GLILD | yes | ITP |  | 23,600 | 51 | 51 | 52 | 0 | no |  | no |
| 6 | Granuloma | lung and diffusely spread lymphadenopathy | yes | ITP, AIHA, collagen colitis |  | 48,875 | 43 | 55 | 55 | 12 | no |  | no |
| 7 | Granuloma | abdominal lymph glands, possibly in spleen | yes |  | Suspected mamma carcinoma (BIRADS 4) | 29,793 | 47 | 51 | 65 | 4 | yes, 2020, v7 | heterozygous NM_012452.2(TNFRSF13B):c.204dupA, p.(Leu69fs) | lymphadenopathy and splenomegaly |
| 8 | Granuloma | lung | no | psoriasis | Basal cell carcinoma skin | 9,614 | 65 | 70 | 70 | 5 | no |  | no |
| 9 | Granuloma | spleen | yes | psoriasis |  | 6,759 | 26 | 35 | 41 | 9 | yes, 2020, v7 | none | no |
| 10 | Granuloma | GLILD, lymphadenopathy cervical and inguinal | no | ITP, AIHA |  | 22,400 | 7 | 16 | 14 | 9 | no |  | lymphadenopathy |
| 11 | Granuloma | suspected GLILD | no | psoriasis |  | 25,089 | 53 | 56 | 56 | 3 | no |  | no |
| 12 | Granuloma | GLILD | yes |  |  | 34,365 | *during twenties (+/- 25)* | 29 | 30 | 4 | yes, 2017 v2.1 | none | no |
| 13 | AI |  |  | ITP, AIHA | Diffuse Large B-cell lymphoma | 3,291 | 9 | 18 | 38 | 9 | no |  | BE, lymphadenopathy |
| 14 | AI |  |  | ITP, AIHA |  | 6,030 | 16 | 15 | 20 | -1 | yes, 2018, v3 | heterozygous NM_012452.2 (TNFRSF13B): c.512T>G, p.(Leu171Arg) | no |
| 15 | AI |  |  | ITP |  | 1,620 | *childhood (+/- 9)* | 16 | 16 | 7 | no |  | no |
| 16 | AI |  |  | SLE |  | 5,613 | 11 | 25 | 26 | 14 | yes, 2016* | none | no |
| 17 | AI |  |  | stiff persons syndrome |  | 3,560 | 60 | 65 | 70 | 5 | no |  | no |
| 18 | AI |  |  | AI gastritis |  | 26,800 | 41 | 41 | 42 | 0 | yes, 2017* | none | no |
| 19 | AI |  |  | ITP, psoriasis |  | 5,400 | 24 | 27 | 27 | 3 | no |  | no |
| 20 | Malignancy |  |  |  | Mamma carcinoma | 2,765 | 59 | 60 | 62 | 1 | no |  | no |
| 21 | Malignancy |  |  |  | Diffuse Large B-cell lymphoma | 3,488 | *during puberty (+/-15)* | 49 | 50 | 34 | yes, 2017 v2.1 | none | no |
| 22 | Malignancy |  |  |  | Kaposi sarcoma | 3,479 | 76 | 76 | 77 | 0 | no |  | no |

**Supplemental table 3**. Characteristics and sIL-2R level of NIC CVID patients. AI: auto-immune complications; GLILD: granulomatous and lymphocytic interstitial lung disease; IBD: irritable bowel syndrome; ITP: immune thrombocytopenia; AIHA: autoimmune hemolytic anemia; BE: bronchiectasis; SLE: systemic lupus erythematosus. *: genetic gene panel according to Picard, C., et al. 2015 [29].

|  | **+p.gran vs IO** | **+p.gran vs sarcoidosis** |
| --- | --- | --- |
| Area | 0.95 | 0.80 |
| Std. Error | 0.03 | 0.06 |
| 95% confidence interval | 0.89 to 1.0 | 0.69 to 0.91 |
| P value | < 0.001 | < 0.05 |
| Cutoff value (pg mL^-1^) | > 6,376 | < 23,223 |
| sensitivity | 91.7% | 91.1% |
| sensitivity 95%CI | 61.5% to 99.8% | 82.6% to 96.4% |
| Specificity | 88.5% | 41.7% |
| specificity 95%CI | 69.9% to 97.6% | 15.1% to 72.3% |
| Likelihood ratio | 7.94 | 1.56 |
| Youden's index | 0.80 | 0.33 |

**Supplementary Table 4. Discriminating capacity sIL-2R for distinguishing between CVID-IO vs CVID+p.granuloma and CVID+p.granuloma vs sarcoidosis**
